# Complete genome sequences of two *Klebsiella pneumoniae* phages from Dakar, Senegal

**DOI:** 10.1128/mra.00047-24

**Published:** 2024-02-20

**Authors:** Issa Ndiaye, Laurent Debarbieux, Moussa Moise Diagne, Ousmane Sow, Abdoulaye Cissé, Bissoume Sambe Ba, Cheikh Fall, Yakhya Dieye, Ndongo Dia, Abdoulaye Seck, Guillaume Constantin de Magny

**Affiliations:** 1 Pole de Microbiologie, Institut Pasteur de Dakar, Dakar, Senegal; 2 Faculté de Médecine, Pharmacie et Odontostomatologie, Université Cheikh Anta Diop, Dakar, Senegal; 3 Laboratoire de Bactériophage, Bactérie, Hôte, Département de Microbiologie, Institut Pasteur Paris, Paris, France; 4 Département de Virologie, Institut Pasteur de Dakar, Dakar, Senegal; 5 WCARO, World Health Organization, Dakar, Senegal; 6 MIVEGEC, Univ. Montpellier, CNRS, IRD, Montpellier, France; 7 MEEDiN, Montpellier Ecology and Evolution of Disease Network, Montpellier, France; Queens College Department of Biology, New York, USA

**Keywords:** bacteriophages, *Klebsiella pneumoniae*, bacteriophage therapy, Healthcare associated infections

## Abstract

Two bacteriophages (phages) of *Klebsiella pneumoniae* were isolated from sewage water collected from Dakar, Senegal. Phage vKpIN17 belongs to the *Przondovirus* genus within the *Autographiviridae* family, with double-stranded DNA genomes, whereas vKpIN18 belongs to the *Webervirus* genus of the *Drexlerviridae* family.

## ANNOUNCEMENT

**Fig 1:**
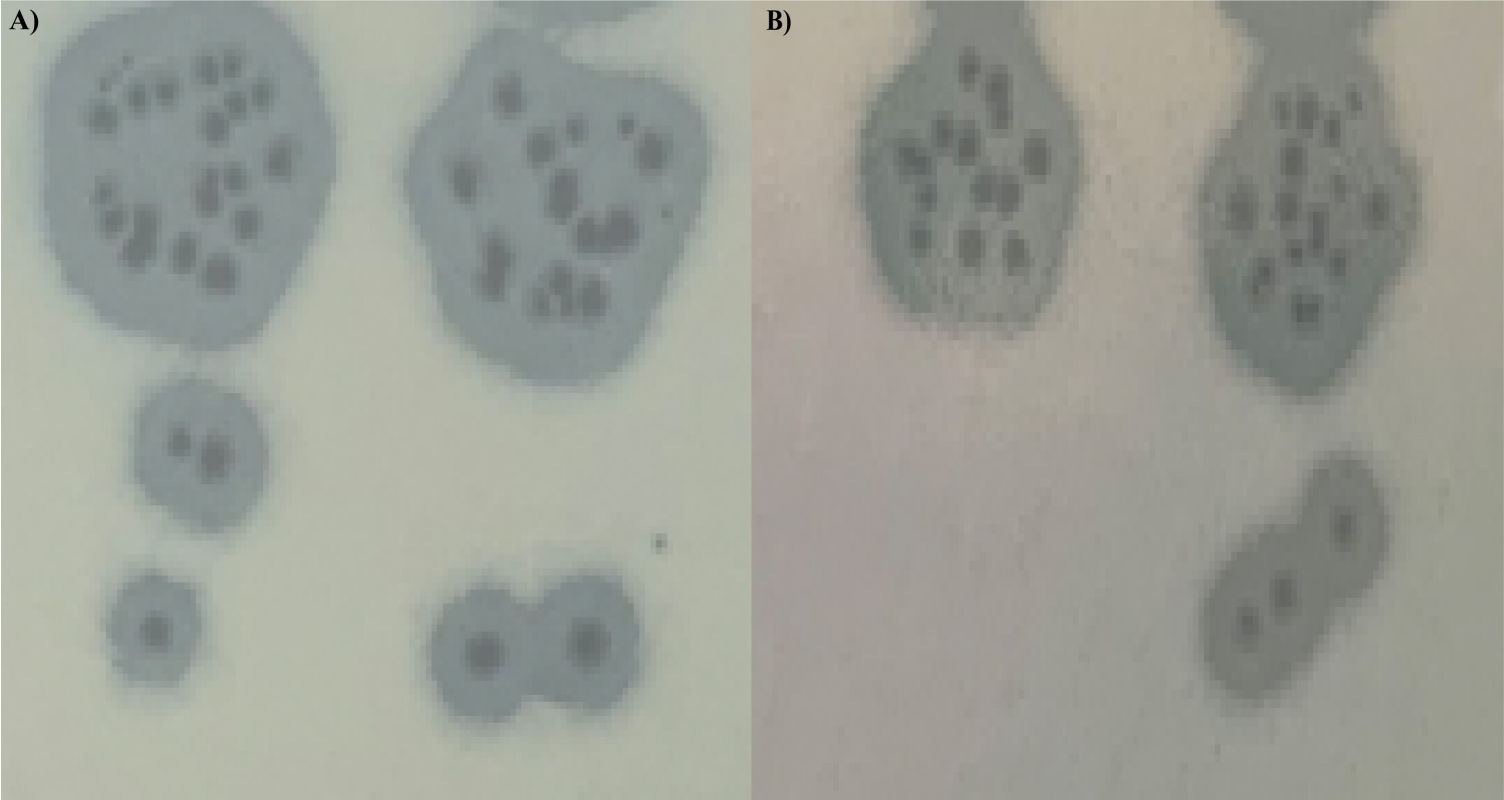
Plaque morphology of phages (**A**) vKpIN17 and (**B**) vKpIN18.

Using a clinical strain of *Klebsiella pneumoniae* KP26 isolated from healthcare-associated infection in a Children’s Hospital Center Albert Royer of Fann in Dakar, two lytic bacteriophages were isolated from two community sewage water (14.695121–17.455776; 14.685763–17.46923) in Dakar, Senegal. Twenty milliliters of sewage sample was centrifugated at 5,000 *g* for 10 min, and then the supernatants were filtered through a 0.22 µm pore size membrane filter. We assessed the filtered supernatants individually for phage presence using double layer agar method (1). Phage plaques were processed by three rounds of purification and amplified as previously described (2). The two phages produced clear plaques with a large halo (Fig. 1).

Genomic DNA was isolated from high titer stocks (>10^9^ PFU/mL). Briefly, 1 mL of phage lysate was treated with 10 µL of DNase I (20 U) and 4 µL of RNase A (20 mg/mL), incubated for 30 min at 37°C, followed by DNA extraction using phenol-chloroform method (2). Library preparations were performed using 1 ng of DNA and Nextera XT DNA library preparation kits (Illumina, San Diego, CA, USA) and executed according to the manufacturer’s protocol. Whole-genome sequencing was performed on Illumina iSeq100 sequencers utilizing the 300-cycle i1 Reagent V2 Kit (Illumina, San Diego, CA, USA).

A total of 299,580 and 323,244 (2 × 150 bp) paired-end reads were generated, respectively, for vKpIN17 and vKpIN18, and quality control was performed with FastQC v0.12.1 (3). Reads were trimmed using trim-galore v0.6.10 (4). The *de novo* assembly was performed using SPAdes v.3.15.5 (5) with careful parameters. Contig coverage and assembly validation were performed with BBMap v 35.85 (6). Furthermore, reads were sorted and indexed using Samtools v1.18 (7) and were submitted to assembly error corrections using Pilon v1.24 (8). Phage termini were identified with PhageTerm (9). Predicted coding sequences (CDS) were annotated using Pharokka v1.3.0 (10). For taxonomic classification, closely related genomes were obtained from the NCBI database.

The vKpIN17 and vKpIN18 genome sizes were 40,702 and 48,639 bp, respectively, with GC contents of 53.35% and 50.4%, and mean coverage of 1,674× and 1,481×. Both phages’ genomes are permutated and feature redundant ends. There are, respectively, 54 and 84 predicted CDS of which 24 (44%) and 48 (57%) are hypothetical proteins for vKpIN17 and vKpIN18. CDS with homology to other known genes encode, among others, structural elements, DNA, RNA and nucleotide metabolism, and host lysis. No genes associated with lysogeny (e.g., integrases), virulence, toxin, transfer RNAs, clustered regularly interspaced short palindromic repeats, or antibiotic resistance were detected within their genomes.

The closest phages of vKpIN17 and vKpIN18 were *Klebsiella* phage K11 (accession: NC_011043.1) (95.99%) and *Klebsiella* phage Kp8 (accession: NC_048700.1) (95.70%), respectively. The phage K11 belongs to the *Przondovirus K11* species, *Przondovirus* genus, and *Autographiviridae* family, while Kp8 belongs to the *Webervirus KLPPOU149* species, *Webervirus* genus, and *Drexlerviridae* family.

We have identified two phages, vKpIN17 and vKpIN18, that show significant promise as candidates for therapeutic applications. However, further characterization is needed to explore their putative therapeutic value.

## Data Availability

The data for the two phages are available in the European Nucleotide Archive (ENA) under the project number PRJEB68306 with accession numbers ERP153263 (vKpIN17) and ERP153263 (vKpIN18) for raw sequences reads and accession numbers GCA_963669465 (vKpIN17) and GCA_963669475 (vKpIN18) for genome sequences.
